# (*E*)-1-(2-Hy­droxy-5-meth­oxy­benzyl­idene)thio­semicarbazide

**DOI:** 10.1107/S1600536811056182

**Published:** 2012-01-11

**Authors:** Amir Adabi Ardakani, Hadi Kargar, Reza Kia, Muhammad Nawaz Tahir

**Affiliations:** a Ardakan Branch, Islamic Azad University, Ardakan, Iran; b Department of Chemistry, Payame Noor University, PO BOX 19395-3697 Tehran, I. R. of IRAN; c X-ray Crystallography Lab., Plasma Physics Research Center, Science and Research Branch, Islamic Azad University, Tehran, IRAN; d Department of Chemistry, Science and Research Branch, Islamic Azad University, Tehran, Iran; e Department of Physics, University of Sargodha, Punjab, Pakistan

## Abstract

In the title mol­ecule, C_9_H_11_N_3_O_2_S, an intra­molecular O—H⋯N hydrogen bond generates an *S*(6) ring motif. In the crystal, mol­ecules are linked *via* pairs of N—H⋯S inter­actions, forming inversion dimers with *R*_2_^2^(8) ring motifs. These dimers are further linked *via* N—H⋯S and N—H⋯O hydrogen bonds, forming a two-dimensional network lying parallel to (100). The crystal structure is further stabilized by inter­molecular π–π inter­actions [centroid–centroid distance = 3.7972 (9) Å].

## Related literature

For hydrogen-bond motifs, see: Bernstein *et al.* (1995 ▶). For background to thio­semicarbazones in coordination chemistry, see: Casas *et al.* (2000 ▶). For their biological applications, see: Maccioni *et al.* (2003 ▶); Ferrari *et al.* (2000 ▶). For related structures, see: Kargar *et al.* (2010*a* ▶,*b* ▶); Adabi Ardakani *et al.* (2012 ▶).
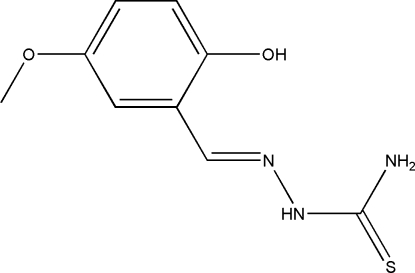


## Experimental

### 

#### Crystal data

C_9_H_11_N_3_O_2_S
*M* *_r_* = 225.27
Monoclinic, 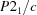

*a* = 7.4878 (2) Å
*b* = 9.9880 (2) Å
*c* = 14.3754 (3) Å
β = 91.846 (1)°
*V* = 1074.55 (4) Å^3^
*Z* = 4
Mo *K*α radiation
μ = 0.29 mm^−1^
*T* = 291 K
0.24 × 0.14 × 0.08 mm

#### Data collection

Bruker SMART APEXII CCD area-detector diffractometer
Absorption correction: multi-scan (*SADABS*; Bruker, 2005 ▶) *T*_min_ = 0.800, *T*_max_ = 0.926
10176 measured reflections
2673 independent reflections
2365 reflections with *I* > 2σ(*I*)
*R*_int_ = 0.016

#### Refinement

*R*[*F* ^2^ > 2σ(*F* ^2^)] = 0.035
*wR*(*F* ^2^) = 0.110
*S* = 1.07
2673 reflections
138 parameters
H-atom parameters constrained
Δρ_max_ = 0.31 e Å^−3^
Δρ_min_ = −0.24 e Å^−3^

### 

Data collection: *APEX2* (Bruker, 2005 ▶); cell refinement: *SAINT* (Bruker, 2005 ▶); data reduction: *SAINT*; program(s) used to solve structure: *SHELXS97* (Sheldrick, 2008 ▶); program(s) used to refine structure: *SHELXL97* (Sheldrick, 2008 ▶); molecular graphics: *SHELXTL* (Sheldrick, 2008 ▶); software used to prepare material for publication: *SHELXTL* and *PLATON* (Spek, 2009 ▶).

## Supplementary Material

Crystal structure: contains datablock(s) global, I. DOI: 10.1107/S1600536811056182/su2361sup1.cif

Structure factors: contains datablock(s) I. DOI: 10.1107/S1600536811056182/su2361Isup2.hkl

Supplementary material file. DOI: 10.1107/S1600536811056182/su2361Isup3.cml

Additional supplementary materials:  crystallographic information; 3D view; checkCIF report

## Figures and Tables

**Table 1:** Hydrogen-bond geometry (Å, °)

*D*—H⋯*A*	*D*—H	H⋯*A*	*D*⋯*A*	*D*—H⋯*A*
O1—H1⋯N1	0.82	1.97	2.6844 (15)	146
N2—H2⋯S1^i^	0.86	2.61	3.3706 (12)	148
N3—H3*A*⋯S1^ii^	0.86	2.66	3.2706 (12)	129
N3—H3*B*⋯O1^iii^	0.86	2.11	2.9604 (17)	172

Symmetry codes: (i) 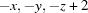; (ii) 
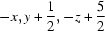
; (iii) 
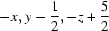
.

**Fig. 1.**
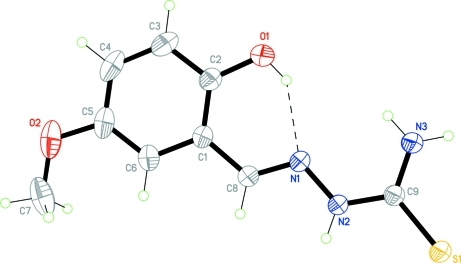
The molecular structure of the title molecule, showing 40% probability displacement ellipsoids and the atomic numbering. The intramolecular O-H···N hydrogen bond is drawn as a dashed line.

**Fig. 2.**
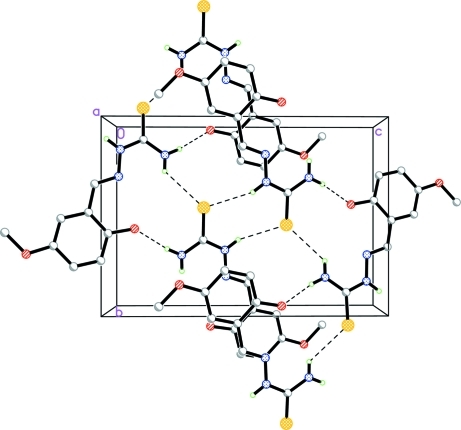
The crystal packing of the title compound, viewed along the a-axis, showing the two-dimensional extended network parallel to (1 0 0). The N—H···S and N—H···O, hydrogen bonds are shown as dashed lines [see Table 1 for details; only the H atoms involved in these interactions are shown].

## supplementary crystallographic information

### Comment

Thiosemicarbazones constitute an important class of N,S donor ligands due to their propensity to react with a wide range of metals (Casas *et al.*, 2000). Thiosemicarbazones exhibit various biological activities and have therefore attracted considerable pharmaceutical interest (Maccioni *et al.*, 2003; Ferrari *et al.*, 2000). We report herein on the synthesis and crystal structure of the title a hydrazone Schiff base compound.

The asymmetric unit of the title compound, Fig. 1, comprises a hydrazone Schiff base ligand. The bond lengths and angles are within the normal ranges and are comparable to those reported for related structures (Kargar *et al.*, 2010**a*,b*; Adabi *et al.*, 2012). An intramolecular O—H···N hydrogen bond (Table 1) generates an S(6) ring motif (Bernstein *et al.*, 1995).

In the crystal, pairs of intermolecular N—H···S hydrogen bonds make inversion dimers with an R^2^_2_(8) ring motif. Intermolecular N—H···S and N—H···O hydrogen bonds link neighbouring molecules into a two-dimensional extended network parallel to (1 0 0). For details of the hydrogen bonding see Table 1, and Fig. 2. The crystal structure is further stabilized by an intermolecular π···π interaction [*Cg*1···*Cg*1^i^ = 3.7972 (9)Å; (i) -x+1, -y+1, -z+2; *Cg*1 is the centroid of ring (C1–C6)].

### Experimental

A mixture of 5-methoxysalicylalehyde (0.01 mol) and hydrazinecarbothioamide (0.01 mol) in 20 ml of ethanol was refluxed for about 2 h. On cooling, the solid separated was filtered and recrystallized from ethanol. Colourless plate-like crystals of the title compound, suitable for X-ray diffraction, were obtained by slow evaporation of a solution in ethanol.

### Refinement

O- and N-bound H atoms were located in a difference Fourier map and were constrained to ride on their parent atoms: O-H = 0.82 Å, N-H = 0.86 Å, with U_iso_(H) = 1.5U_eq_(O) and 1.2U_eq_(N). The C-bound H-atoms were included in calculated positions and treated as riding atoms: C—H = 0.93 and 0.96 Å for CH and CH_3_ H-atoms, respectively, with U_iso_ (H) = k × U_eq_(C), where k = 1.5 for CH_3_ H-atoms, and k = 1.2 for all other H-atoms.

### Crystal data

**Table:**

C_9_H_11_N_3_O_2_S	*F*(000) = 472
*M**_r_* = 225.27	*D*_x_ = 1.392 Mg m^−^^3^
Monoclinic, *P*2_1_/*c*	Mo *K*α radiation, λ = 0.71073 Å
Hall symbol: -P 2ybc	Cell parameters from 2750 reflections
*a* = 7.4878 (2) Å	θ = 2.4–27.5°
*b* = 9.9880 (2) Å	µ = 0.29 mm^−^^1^
*c* = 14.3754 (3) Å	*T* = 291 K
β = 91.846 (1)°	Plate, colourless
*V* = 1074.55 (4) Å^3^	0.24 × 0.14 × 0.08 mm
*Z* = 4	

### Data collection

**Table:**

Bruker SMART APEXII CCD area-detector diffractometer	2673 independent reflections
Radiation source: fine-focus sealed tube	2365 reflections with *I* > 2σ(*I*)
graphite	*R*_int_ = 0.016
φ and ω scans	θ_max_ = 28.4°, θ_min_ = 2.5°
Absorption correction: multi-scan (*SADABS*; Bruker, 2005)	*h* = −9→9
*T*_min_ = 0.800, *T*_max_ = 0.926	*k* = −13→13
10176 measured reflections	*l* = −17→19

### Refinement

**Table:**

Refinement on *F*^2^	Primary atom site location: structure-invariant direct methods
Least-squares matrix: full	Secondary atom site location: difference Fourier map
*R*[*F*^2^ > 2σ(*F*^2^)] = 0.035	Hydrogen site location: inferred from neighbouring sites
*wR*(*F*^2^) = 0.110	H-atom parameters constrained
*S* = 1.07	*w* = 1/[σ^2^(*F*_o_^2^) + (0.0594*P*)^2^ + 0.2733*P*] where *P* = (*F*_o_^2^ + 2*F*_c_^2^)/3
2673 reflections	(Δ/σ)_max_ = 0.001
138 parameters	Δρ_max_ = 0.31 e Å^−^^3^
0 restraints	Δρ_min_ = −0.24 e Å^−^^3^

### Special details

**Table:**

Geometry. All esds (except the esd in the dihedral angle between two l.s. planes) are estimated using the full covariance matrix. The cell esds are taken into account individually in the estimation of esds in distances, angles and torsion angles; correlations between esds in cell parameters are only used when they are defined by crystal symmetry. An approximate (isotropic) treatment of cell esds is used for estimating esds involving l.s. planes.
Refinement. Refinement of F^2^ against ALL reflections. The weighted R-factor wR and goodness of fit S are based on F^2^, conventional R-factors R are based on F, with F set to zero for negative F^2^. The threshold expression of F^2^ > 2sigma(F^2^) is used only for calculating R-factors(gt) etc. and is not relevant to the choice of reflections for refinement. R-factors based on F^2^ are statistically about twice as large as those based on F, and R- factors based on ALL data will be even larger.

### Fractional atomic coordinates and isotropic or equivalent isotropic displacement parameters (Å^2^)

**Table:**

	*x*	*y*	*z*	*U*_iso_*/*U*_eq_	
C1	0.26170 (16)	0.48062 (13)	0.97372 (9)	0.0346 (3)	
C2	0.24057 (17)	0.58273 (14)	1.03826 (10)	0.0386 (3)	
C3	0.2907 (2)	0.71257 (15)	1.01635 (13)	0.0510 (4)	
H3	0.2758	0.7812	1.0591	0.061*	
C4	0.3626 (2)	0.73967 (16)	0.93147 (14)	0.0573 (4)	
H4	0.3962	0.8268	0.9175	0.069*	
C5	0.3857 (2)	0.63922 (17)	0.86653 (12)	0.0512 (4)	
C6	0.33436 (19)	0.51057 (16)	0.88716 (10)	0.0438 (3)	
H6	0.3478	0.4429	0.8435	0.053*	
C7	0.4842 (3)	0.5753 (3)	0.71703 (14)	0.0797 (7)	
H7A	0.5574	0.5054	0.7437	0.120*	
H7B	0.5415	0.6124	0.6641	0.120*	
H7C	0.3701	0.5392	0.6978	0.120*	
C8	0.21137 (17)	0.34293 (13)	0.99115 (9)	0.0365 (3)	
H8	0.2270	0.2798	0.9445	0.044*	
C9	0.02639 (18)	0.11834 (12)	1.14708 (9)	0.0353 (3)	
N1	0.14624 (14)	0.30443 (10)	1.06812 (8)	0.0349 (2)	
N2	0.10299 (16)	0.17032 (11)	1.07211 (8)	0.0388 (3)	
H2	0.1255	0.1192	1.0258	0.047*	
N3	0.0098 (2)	0.19397 (12)	1.22149 (8)	0.0507 (3)	
H3A	0.0478	0.2752	1.2214	0.061*	
H3B	−0.0390	0.1620	1.2701	0.061*	
O1	0.17090 (17)	0.56061 (11)	1.12351 (8)	0.0514 (3)	
H1	0.1484	0.4807	1.1292	0.077*	
O2	0.4601 (2)	0.67718 (16)	0.78420 (11)	0.0777 (4)	
S1	−0.04238 (6)	−0.04281 (3)	1.14369 (3)	0.04739 (14)	

### Atomic displacement parameters (Å^2^)

**Table:**

	*U*^11^	*U*^22^	*U*^33^	*U*^12^	*U*^13^	*U*^23^
C1	0.0324 (6)	0.0336 (6)	0.0378 (6)	−0.0011 (5)	0.0011 (5)	0.0040 (5)
C2	0.0356 (6)	0.0346 (6)	0.0455 (7)	0.0003 (5)	0.0019 (5)	0.0011 (5)
C3	0.0486 (8)	0.0323 (7)	0.0722 (10)	−0.0006 (6)	0.0028 (7)	−0.0008 (7)
C4	0.0489 (8)	0.0387 (7)	0.0844 (12)	−0.0041 (6)	0.0022 (8)	0.0213 (8)
C5	0.0422 (7)	0.0543 (9)	0.0573 (9)	−0.0021 (6)	0.0051 (6)	0.0232 (7)
C6	0.0430 (7)	0.0474 (8)	0.0411 (7)	−0.0017 (6)	0.0048 (5)	0.0072 (6)
C7	0.0654 (11)	0.123 (2)	0.0515 (10)	0.0004 (12)	0.0163 (9)	0.0260 (12)
C8	0.0411 (6)	0.0329 (6)	0.0356 (6)	−0.0024 (5)	0.0041 (5)	−0.0009 (5)
C9	0.0440 (6)	0.0293 (6)	0.0326 (6)	0.0004 (5)	0.0021 (5)	0.0016 (5)
N1	0.0401 (5)	0.0285 (5)	0.0362 (5)	−0.0025 (4)	0.0027 (4)	0.0010 (4)
N2	0.0540 (6)	0.0282 (5)	0.0346 (5)	−0.0040 (4)	0.0091 (5)	−0.0012 (4)
N3	0.0834 (9)	0.0347 (6)	0.0347 (6)	−0.0109 (6)	0.0138 (6)	−0.0034 (5)
O1	0.0678 (7)	0.0401 (5)	0.0473 (6)	−0.0046 (5)	0.0163 (5)	−0.0072 (4)
O2	0.0770 (9)	0.0822 (10)	0.0750 (9)	−0.0103 (7)	0.0193 (7)	0.0389 (8)
S1	0.0754 (3)	0.02908 (19)	0.0382 (2)	−0.00904 (14)	0.01049 (17)	0.00096 (12)

### Geometric parameters (Å, °)

**Table:**

C1—C2	1.3912 (19)	C7—H7A	0.9600
C1—C6	1.4063 (18)	C7—H7B	0.9600
C1—C8	1.4499 (17)	C7—H7C	0.9600
C2—O1	1.3653 (17)	C8—N1	1.2824 (16)
C2—C3	1.389 (2)	C8—H8	0.9300
C3—C4	1.376 (2)	C9—N3	1.3187 (17)
C3—H3	0.9300	C9—N2	1.3415 (16)
C4—C5	1.385 (3)	C9—S1	1.6901 (13)
C4—H4	0.9300	N1—N2	1.3797 (14)
C5—C6	1.376 (2)	N2—H2	0.8600
C5—O2	1.3774 (19)	N3—H3A	0.8600
C6—H6	0.9300	N3—H3B	0.8600
C7—O2	1.418 (3)	O1—H1	0.8200
			
C2—C1—C6	119.32 (13)	H7A—C7—H7B	109.5
C2—C1—C8	122.98 (12)	O2—C7—H7C	109.5
C6—C1—C8	117.70 (12)	H7A—C7—H7C	109.5
O1—C2—C3	117.94 (13)	H7B—C7—H7C	109.5
O1—C2—C1	122.33 (12)	N1—C8—C1	122.80 (12)
C3—C2—C1	119.72 (13)	N1—C8—H8	118.6
C4—C3—C2	120.06 (15)	C1—C8—H8	118.6
C4—C3—H3	120.0	N3—C9—N2	118.93 (12)
C2—C3—H3	120.0	N3—C9—S1	122.17 (10)
C3—C4—C5	121.07 (14)	N2—C9—S1	118.89 (10)
C3—C4—H4	119.5	C8—N1—N2	115.04 (11)
C5—C4—H4	119.5	C9—N2—N1	121.08 (11)
C6—C5—O2	124.45 (17)	C9—N2—H2	119.5
C6—C5—C4	119.29 (14)	N1—N2—H2	119.5
O2—C5—C4	116.26 (15)	C9—N3—H3A	120.0
C5—C6—C1	120.53 (15)	C9—N3—H3B	120.0
C5—C6—H6	119.7	H3A—N3—H3B	120.0
C1—C6—H6	119.7	C2—O1—H1	109.5
O2—C7—H7A	109.5	C5—O2—C7	116.83 (15)
O2—C7—H7B	109.5		
			
C6—C1—C2—O1	179.97 (12)	C2—C1—C6—C5	0.6 (2)
C8—C1—C2—O1	0.2 (2)	C8—C1—C6—C5	−179.64 (13)
C6—C1—C2—C3	0.2 (2)	C2—C1—C8—N1	−1.2 (2)
C8—C1—C2—C3	−179.54 (13)	C6—C1—C8—N1	179.03 (12)
O1—C2—C3—C4	179.62 (14)	C1—C8—N1—N2	178.93 (11)
C1—C2—C3—C4	−0.6 (2)	N3—C9—N2—N1	−7.2 (2)
C2—C3—C4—C5	0.2 (2)	S1—C9—N2—N1	173.92 (10)
C3—C4—C5—C6	0.6 (2)	C8—N1—N2—C9	−177.01 (12)
C3—C4—C5—O2	−179.49 (14)	C6—C5—O2—C7	0.0 (2)
O2—C5—C6—C1	179.10 (14)	C4—C5—O2—C7	−179.90 (16)
C4—C5—C6—C1	−1.0 (2)		

### Hydrogen-bond geometry (Å, °)

**Table:**

*D*—H···*A*	*D*—H	H···*A*	*D*···*A*	*D*—H···*A*
O1—H1···N1	0.82	1.97	2.6844 (15)	146
N2—H2···S1^i^	0.86	2.61	3.3706 (12)	148
N3—H3A···S1^ii^	0.86	2.66	3.2706 (12)	129
N3—H3B···O1^iii^	0.86	2.11	2.9604 (17)	172

Symmetry codes: (i) −*x*, −*y*, −*z*+2; (ii) −*x*, *y*+1/2, −*z*+5/2; (iii) −*x*, *y*−1/2, −*z*+5/2.

## References

[bb1] Adabi Ardakani, A., Kargar, H., Kia, R. & Tahir, M. N. (2012). *Acta Cryst.* E**68**, o340–o341. 10.1107/S1600536812000487 PMC3275025 22346970

[bb2] Bernstein, J., Davis, R. E., Shimoni, L. & Chang, N.-L. (1995). *Angew. Chem. Int. Ed. Engl.* **34**, 1555–1573.

[bb3] Bruker (2005). *APEX2*, *SAINT* and *SADABS* Bruker AXS Inc., Madison, Wisconsin, USA.

[bb4] Casas, J. S., Garcia-Tasende, M. S. & Sordo, J. (2000). *Coord. Chem. Rev.* **209**, 197–261.

[bb5] Ferrari, M. B., Capacchi, S., Reffo, G., Pelosi, G., Tarasconi, P., Albertini, R., Pinelli, S. & Lunghi, P. (2000). *J. Inorg. Biochem.* **81**, 89–97. 10.1016/s0162-0134(00)00087-8 11001436

[bb6] Kargar, H., Kia, R., Akkurt, M. & Büyükgüngör, O. (2010*a*). *Acta Cryst.* E**66**, o2999. 10.1107/S1600536810043357 PMC3009273 21589160

[bb7] Kargar, H., Kia, R., Akkurt, M. & Büyükgüngör, O. (2010*b*). *Acta Cryst.* E**66**, o2981. 10.1107/S1600536810043448 PMC3009088 21589147

[bb8] Maccioni, E., Cardia, M. C., Distinto, S., Bonsignore, L. & De Logu, A. (2003). *Farmaco* **58**, 951–959. 10.1016/S0014-827X(03)00154-X 13679191

[bb9] Sheldrick, G. M. (2008). *Acta Cryst.* A**64**, 112–122. 10.1107/S0108767307043930 18156677

[bb10] Spek, A. L. (2009). *Acta Cryst.* D**65**, 148–155. 10.1107/S090744490804362X PMC2631630 19171970

